# Photoinactivation of Yeast and Biofilm Communities of *Candida albicans* Mediated by ZnTnHex-2-PyP^4+^ Porphyrin

**DOI:** 10.3390/jof8060556

**Published:** 2022-05-25

**Authors:** Sueden O. Souza, Bruno L. Raposo, José F. Sarmento-Neto, Júlio S. Rebouças, Danielle P. C. Macêdo, Regina C. B. Q. Figueiredo, Beate S. Santos, Anderson Z. Freitas, Paulo E. Cabral Filho, Martha S. Ribeiro, Adriana Fontes

**Affiliations:** 1 Departamento de Biofísica e Radiobiologia, Universidade Federal de Pernambuco, Recife 50670-901, PE, Brazil; bruno.raposo@ufpe.br (B.L.R.); paulo.euzebio@ufpe.br (P.E.C.F.); 2 Departamento de Química, Universidade Federal da Paraíba, João Pessoa 58051-900, PB, Brazil; ferreira.system@gmail.com (J.F.S.-N.); jsreboucas@quimica.ufpb.br (J.S.R.); 3 Departamento de Ciências Farmacêuticas, Universidade Federal de Pernambuco, Recife 50740-520, PE, Brazil; danielle.cerqueira@ufpe.br (D.P.C.M.); beate.santos@ufpe.br (B.S.S.); 4 Departamento de Microbiologia, Instituto Aggeu Magalhães—Fundação Oswaldo Cruz (IAM-FIOCRUZ), Recife 50740-465, PE, Brazil; rcbqf01@gmail.com; 5 Centro de Lasers e Aplicações, Instituto de Pesquisas Energéticas e Nucleares (IPEN-CNEN), São Paulo 05508-000, SP, Brazil; freitas.az.ipen@gmail.com (A.Z.F.); marthasr@usp.br (M.S.R.)

**Keywords:** antimicrobial photodynamic inactivation, blue light, fungi, photodynamic therapy, Zn(II) porphyrin

## Abstract

*Candida albicans* is the main cause of superficial candidiasis. While the antifungals available are defied by biofilm formation and resistance emergence, antimicrobial photodynamic inactivation (aPDI) arises as an alternative antifungal therapy. The tetracationic metalloporphyrin Zn(II) *meso*-tetrakis(*N*-n-hexylpyridinium-2-yl)porphyrin (ZnTnHex-2-PyP^4+^) has high photoefficiency and improved cellular interactions. We investigated the ZnTnHex-2-PyP^4+^ as a photosensitizer (PS) to photoinactivate yeasts and biofilms of *C. albicans* strains (ATCC 10231 and ATCC 90028) using a blue light-emitting diode. The photoinactivation of yeasts was evaluated by quantifying the colony forming units. The aPDI of ATCC 90028 biofilms was assessed by the MTT assay, propidium iodide (PI) labeling, and scanning electron microscopy. Mammalian cytotoxicity was investigated in Vero cells using MTT assay. The aPDI (4.3 J/cm^2^) promoted eradication of yeasts at 0.8 and 1.5 µM of PS for ATCC 10231 and ATCC 90028, respectively. At 0.8 µM and same light dose, aPDI-treated biofilms showed intense PI labeling, about 89% decrease in the cell viability, and structural alterations with reduced hyphae. No considerable toxicity was observed in mammalian cells. Our results introduce the ZnTnHex-2-PyP^4+^ as a promising PS to photoinactivate both yeasts and biofilms of *C. albicans*, stimulating studies with other *Candida* species and resistant isolates.

## 1. Introduction

Superficial fungal infections have been estimated to afflict 20–25% of the global population, and *Candida albicans* appears as the main etiological agent of mucosal conditions [1,2]. *C. albicans* is a major opportunistic pathogen of humans, and the main causative species of candidiasis. This fungus can be normally found as a commensal in the human oral and vaginal mucosa, gastrointestinal tract, and skin, but it can overgrow and cause infection, especially under immunocompromising conditions [2,3]. Oral candidiasis afflicts about 20% of cancer patients and up to 31% of individuals with acquired immunodeficiency syndrome (AIDS) [4], and vulvovaginal candidiasis affects approximately 75% of women globally [5,6].

*C. albicans* infection is aggravated by biofilm production, in which fungal cells in the forms of yeasts, pseudohyphae, and hyphae are found embedded in an extracellular matrix (ECM) [3]. Cells within biofilms have reduced metabolism, and are typically more resistant than planktonic yeasts to antifungal therapies [7]. Furthermore, the antifungal drug repertoire available to treat candidiasis faces challenges relating to fungistatic rather than fungicidal action (azoles), high toxicity (amphotericin B), intravenous administration (echinocandins), and increasing emergence of resistance [8]. The dangerous of antifungal resistance and its impact on the public health worldwide must be recognized, and the development of new antifungal technologies has been fomented to afford the control of fungal infections [9].

In this scenario, antimicrobial photodynamic inactivation (aPDI) has been reported as a promising antifungal method. In aPDI, the activation of a photosensitizer (PS) by light, at a suitable wavelength, leads to oxidative stress-mediated cell death [10]. Due to the generalized effect of reactive oxygen species (ROS) across multiple subcellular components, aPDI has not yet been associated with resistance selection, and its fungicidal action can also eliminate microorganisms resistant to conventional pharmaceuticals [11,12]. Among other advantages, aPDI is expected to provide a quick, localized, and minimally invasive topical treatment, also minimizing the adverse effects that may derive from conventional pharmacotherapy [13]. Furthermore, the return of normal tissue function has been reported following the clinical application of aPDI [14].

Different PSs have been reported for aPDI of *C. albicans*, including methylene blue, curcumin, and porphyrins [15,16,17,18,19]. While these studies demonstrate the potential of aPDI as an antimicrobial method against *C. albicans*, the challenges associated with biofilm eradication is still a concern. Moreover, to allow the development of improved aPDI protocols, the search for adequate PSs is paramount. Suitable PS candidates should present high efficiency at low concentrations, and require mild aPDI parameters, with shorter incubation and irradiation times, as a means to reduce unwanted photoeffects on healthy host tissues [20,21].

Porphyrins and their diamagnetic metallo-derivatives are structurally diverse tetrapyrrole compounds widely explored as PSs, especially in anticancer therapy [22]. Zn(II) chelation in the porphyrin ring may bring advantages over their free-base analogues, such as enhanced singlet oxygen quantum yield and triplet state lifetime, improved solubility and stability, and increased cellular uptake [10,23,24]. As cationic PSs tend to have greater affinity for the highly negative surface of microbial cells, Zn(II) porphyrins with a variety of *meso* and/or *beta* substituents can be designed to control the compound charge and modulate lipophilicity, improving uptake by target cells and clearance from the body [10,25,26].

The aPDI mediated by water-soluble Zn(II) *N*-alkylpyridiniumporphyrins has been explored for a variety of microorganisms, such as bacteria, parasites, and fungi [21,25,27,28,29]. In a previous study, our group reported the susceptibility of *C. albicans* yeasts to aPDI mediated by the cationic water-soluble, Zn(II) *meso*-tetrakis(*N*-ethylpyridinium-2-yl)porphyrin (ZnTE-2-PyP^4+^; ZnP ethyl) [29]. We have also obtained promising results in aPDI against *Leishmania brasiliensis*, in studies applying ZnP ethyl [28]. The performance of aPDI on *Leishmania* spp. was still better when the more lipophilic analogue Zn(II) *meso*-tetrakis(*N*-n-hexylpyridinium-2-yl)porphyrin (ZnTnHex-2-PyP^4+^; ZnP hexyl) was applied [21]. Additionally, the ZnP hexyl analogue showed high chemical stability against demetallation and solvolysis in acids and simulated biological fluids [21]. Moghnie et al. [25], using a *Saccharomyces cerevisiae* cell model, also demonstrated that ZnP hexyl presented improved photoefficiency (with minimal dark toxicity) and enhanced cellular interaction as compared to its more hydrophilic analogues. All these features make ZnP hexyl an attractive PS for aPDI of *C. albicans*, especially considering the challenges imposed by biofilms.

The present study introduces the anti-*Candida* action of ZnP hexyl for the first time and aims to inspire future research in this topic. Furthermore, while it is important to discover new antimicrobial compounds, advances within the field of photodynamic therapy (PDT) also include the improvement of photodynamic parameters, seeking to reduce the incubation time, as well as the photosensitizer concentration and light dose. Therefore, in the present study, we describe a promising protocol exploring ZnP hexyl-mediated aPDI for inactivation of yeast and biofilm communities of *C. albicans*.

## 2. Materials and Methods

### 2.1. C. albicans Strains and Growth Conditions

Two strains of *C. albicans* were used in this study. ATCC 10231 strain, initially frozen at −80 °C, was thawed and cultured in Sabouraud Dextrose Agar (SDA, HIMEDIA, Thane, India). ATCC 90028 strain was already maintained in SDA at 4 °C. One loop-full of culture was dispersed in 4 mL of Sabouraud Dextrose Broth (Neogen, Lansing, MI, USA) and incubated at 37 °C for 18 h. The yeasts were centrifuged (580× *g* for 5 min) and washed twice with 1 × phosphate-buffered saline (PBS). Then, cells were resuspended to prepare the inoculum to a final concentration of ~1 × 10^7^ colony-forming units per milliliter (CFU/mL), adjusted using the optical density at 540 nm (OD_540_) in a spectrophotometer (µQuant, BioTek, Santa Clara, CA, USA), which was corroborated by cell counting in a Neubauer chamber.

### 2.2. Biofilm Formation

Upon standardization of biofilm production, it was observed that the ATCC 10231 isolate did not have the ability to produce a robust biofilm. Thus, the biofilm-producing strain ATCC 90028 was incorporated to this study following assessment of aPDI on its yeast forms. The 18 h culture of *C. albicans* ATCC 90028 was harvested (580× *g* for 5 min) and washed twice with PBS, and the yeast suspension was diluted in 11835 RPMI 1640 (Gibco, no phenol red, supplemented with 20 mM HEPES) to a final concentration of ~5 × 10^7^ CFU/mL, using the OD_540_ (µQuant, BioTek), also corroborated by cell counting in a Neubauer chamber. The volume of 100 µL of *C. albicans* inoculum was added to wells of a 96-well plate (K12-096, Kasvi, São José do Pinhais, Brazil) and incubated at 37 °C for 90 min with constant rotation at 75 rpm (TE-424, Tecnal, Piracicaba, Brazil). The wells were gently washed twice with 200 µL of PBS and replenished with 200 µL of RPMI 1640. The plates were incubated at 37 °C for 48 h to allow biofilm formation and maturation. Two plates with biofilms were prepared for each assay, one for the irradiated groups and another for the control and dark groups.

### 2.3. Photoinactivation of C. albicans

The photosensitizer Zn(II) *meso*-tetrakis(*N*-n-hexylpyridinium-2-yl)porphyrin (ZnP hexyl) was synthesized as the chloride salt [21] and characterized [21,30,31] as previously reported. The concentrations of all ZnP hexyl stock solutions were determined spectrophotometrically using published molar absorptivity value of the Soret band at 427 nm (ε_427 nm_ = 436,516 cm^−1^ M^−1^) [30]. For aPDI of *C. albicans* yeasts, 100 µL of fungal suspension were added to wells of a 96-well plate and incubated with 100 µL of PBS (light and control groups) or ZnP hexyl (dark and aPDI groups) for 10 min (pre-irradiation time—PIT). For ATCC 10231 yeasts, the evaluated groups were: (1) control—no PS nor irradiation; (2) light—irradiation in absence of PS; (3) dark—PS at 1.5 µM, without irradiation; (4) aPDI—PS (0.15–1.5 µM) + irradiation. Light and aPDI samples were irradiated for 3 min using a light source (LEDbox, Biolambda, São Paulo, Brazil) at 410 ± 10 nm (ZnP hexyl has absorption maximum at 427 nm), with irradiance set to 24.1 mW/cm^2^ (light dose = 4.3 J/cm^2^). Control and dark groups were kept protected from light for the same amount of time. The aPDI effect on ATCC 90028 yeasts was evaluated based on the most efficient PS concentration found for the strain ATCC 10231. Following this procedure, the yeast suspensions were serially diluted in PBS and 10 µL of each dilution were added to a Petri dish with SDA and incubated for 24 h for CFU counting, as described by Jett et al. [32]. The CFU counts were adjusted with their corresponding dilution factor and converted to base ten logarithms (log_10_) before plotting the data. At least three independent experiments were performed with two replicates per group.

For aPDI of *C. albicans* biofilms, each well containing biofilm was gently washed twice with 200 µL of PBS, followed by incubation with 200 µL of either ZnP hexyl or PBS for 10 min. The experimental groups included: (1) control—no PS nor irradiation; (2) light —irradiation only; (3) dark—PS at 1.5 µM in the dark; (4) aPDI—PS at 0.8 µM and irradiation. The light source, irradiation time, and irradiance were the same as used for yeasts. One of the plates with biofilms was kept in the dark (control and dark groups). The effect of aPDI was assessed by the 3-(4,5-dimethylthiazol-2-yl)-2,5-diphenyltetrazolium bromide (MTT) assay. Immediately after aPDI, wells were gently washed twice with 200 µL of PBS, followed by the addition of 180 µL of 11835 RPMI 1640 (Gibco, Thermo Fisher Scientific, Waltham, MA, USA) and 20 µL of MTT (Sigma-Aldrich, Burlington, MA, USA) 5 mg/mL, and the plates were incubated in the dark for 5 h at 37 °C. The liquid was removed from each well, 200 µL of dimethyl sulfoxide (DMSO) was added, and the plates were kept in the dark, under gentle shaking (GyroMini, Labnet, Edison, NJ, USA), for 15 min to allow the formazan crystals to dissolve. After thorough homogenization, 100 µL of the system was transferred to a new flat bottom 96-well plate for absorbance reading at 570 nm (µQuant, BioTek). Four independent experiments were performed with at least two replicates per group.

### 2.4. Cell Labeling and Confocal Microscopy

The photodynamic effect on *C. albicans* biofilms was also analyzed by propidium iodide (PI) staining. Briefly, *C. albicans* biofilms were grown in cell culture imaging dishes (Greiner Bio-One, 627975) according to the protocols described for biofilm growth, and aPDI was performed as previously described. Following treatment, the samples were gently washed with PBS to remove ZnP hexyl excess, and 1 µg/mL PI (V13245, Invitrogen, Thermo Fisher Scientific, Waltham, MA, USA) was added to cover each biofilm sample. After incubation with PI in the dark for 15 min, the biofilms were washed, and kept in the kit-specific buffer for analyses. An Olympus FV1000 confocal microscope was used to observe the samples at a 40×/NA = 0.95 objective, under excitation at 473 nm. Even though this wavelength cannot efficiently excite the fluorescence of ZnP hexyl, the PI fluorescence was collected at 715/50 nm to avoid detection of the porphyrin. Representative images from three randomly selected positions were acquired for each sample.

### 2.5. Scanning Electron Microscopy

Scanning electron microscopy (SEM) analyses were also carried out to observe the effects of aPDI on *C. albicans* biofilms. Biofilms were grown on cropped bottoms of 24-well plates. The biofilm growth conditions for SEM were otherwise identical to the protocol previously described above. Following aPDI, the biofilms were processed following the protocol previously reported by Aliança et al. [33], and observed under a scanning electron microscope (JSM-5600 LV, JEOL, Akishima, Tokyo, Japan). Each biofilm sample was observed at multiple fields and representative images were acquired.

### 2.6. Cytotoxicity on Mammalian Cells

The cytotoxicity of the photodynamic treatment was analyzed on Vero cells (ATCC CCL-81) by the MTT assay. Briefly, 200 µL of cells (1 × 10^5^ cell/mL) in RPMI 1640 supplemented with 10% fetal bovine serum (FBS), 100 mg/mL streptomycin, and 100 units/mL penicillin (Sigma-Aldrich) were added to the wells of a 96-well plate and cultured overnight at 37 °C and 5% CO_2_. The consumed media was gently removed and replaced with either PBS or ZnP hexyl. The PS concentrations in the photodynamic treatment were 0.8 and 1.5 µM, and the highest concentration was included in the dark group. The other groups (control and light) and the photodynamic parameters (PIT and light dose) were the same as described for *C. albicans*. Following treatment, cells were washed and 180 µL of FBS-free 11835 RPMI 1640 (Gibco, no phenol red, supplemented with 20 mM HEPES) and 20 µL of MTT 5 mg/mL were added to each well. The plates were incubated for 4 h at 37 °C and 5% CO_2_. The liquid was carefully removed from the wells, 200 µL of DMSO was added to each well, and the plates were kept in the dark for 15 min, under gentle rotation (GyroMini, Labnet). The content of each well was homogenized by pipetting and the OD_570_ was measured (µQuant, BioTek). Four independent experiments were performed with two replicates per group.

### 2.7. Statistical Analysis

The experimental data were analyzed through the Mann–Whitney test using the software GraphPad Prism 7.04. The significance level was set as *p* < 0.05.

## 3. Results

### 3.1. Photoinactivation Effect on Yeast Cells

**Figure 1:**
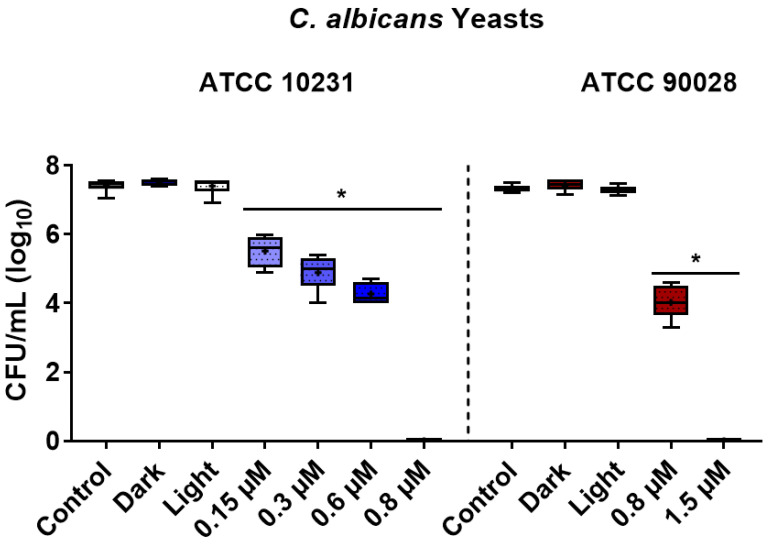
Box plots of photoinactivation of *C. albicans* yeasts. Two different strains were assessed, ATCC 10231 and ATCC 90028. Control: untreated group; dark: yeasts incubated with 1.5 µM of ZnP hexyl without irradiation; light: yeasts irradiated only (4.3 J/cm^2^). The values on the ‘x’ axis indicate the concentrations of ZnP hexyl for different aPDI groups. Differences between groups were analyzed by the Mann–Whitney test and considered significant at *p* < 0.05. The results are expressed as log_10_ of the CFU/mL. At least three independent experiments were performed. *: *p* < 0.05 compared to control.

In the present study, ZnP hexyl was employed as a PS to evaluate the photoinactivation of two strains of *C. albicans*. Yeast cells treated with ZnP hexyl in the dark (1.5 µM, 10 min PIT) or light alone (4.3 J/cm^2^) did not induce noteworthy changes on cell survival (Figure 1). Using the same PIT and light dose (10 min, 4.3 J/cm^2^), aPDI with increasing ZnP hexyl concentrations induced progressive inactivation of fungi. For ATCC 10231, a ~2 log_10_ reduction was observed at the lowest concentration tested, 0.15 µM, and complete eradication was achieved at 0.8 µM (Figure 1). For the strain ATCC 90028, a reduction of 3.3 log_10_ was obtained at 0.8 µM, and complete eradication was observed at 1.5 µM (Figure 1).

### 3.2. Photoinactivation Effect on Biofilms

**Figure 2:**
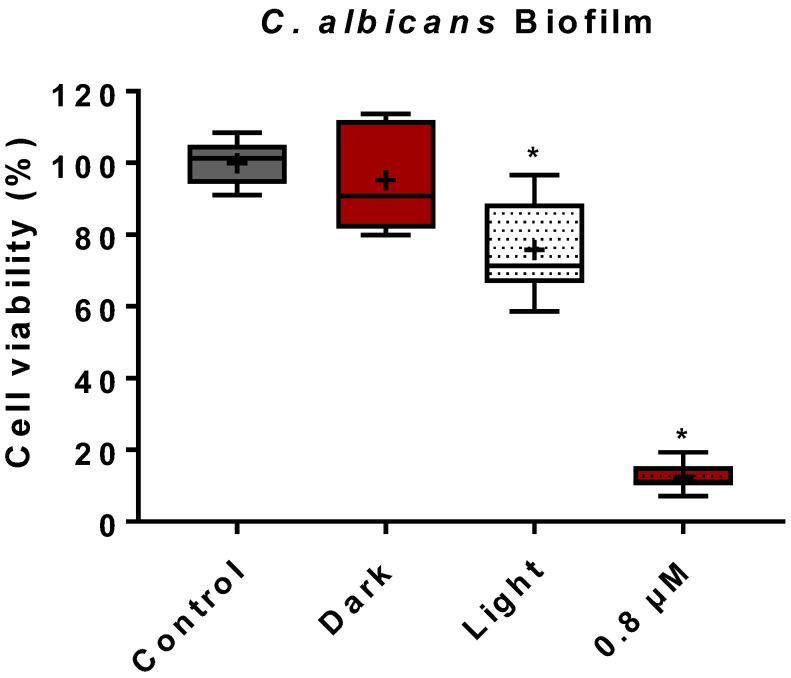
Box plot of cell viability of *C. albicans* ATCC 90028 biofilms assessed through MTT assay following treatment. Control: untreated biofilms; dark: biofilms incubated with 1.5 µM of ZnP hexyl without irradiation; light: biofilms irradiated only (4.3 J/cm^2^). aPDI was performed for biofilms incubated with 0.8 µM of ZnP hexyl for 10 min followed by irradiation (4.3 J/cm^2^). The results were expressed in relation to untreated biofilms. Differences between groups were analyzed by the Man–Whitney test and considered significant at *p* < 0.05. At least three independent experiments were performed. *: *p* < 0.05 compared to control.

Following the establishment of an aPDI protocol for *C. albicans* planktonic cells, our study progressed to assess the more complex and challenging biofilm forms. Compared to the control group (no treatment), the treatment of *C. albicans* biofilms with ZnP hexyl (1.5 µM) in the dark had no significant effect on cell viability (Figure 2). The treatment of biofilms with light alone (4.3 J/cm^2^) induced a reduction of about 30% in cell viability (Figure 2). This effect was still discreet when compared with the reduction of ~89% in cell viability promoted by aPDI mediated by ZnP hexyl (0.8 µM) on biofilms (Figure 2). Furthermore, preliminary results from this study using aPDI with a higher PS concentration (unpublished data), i.e., 2.4 µM, showed further fungal inhibition by 96%, which was a discreet improvement from the 0.8 µM PS concentration.

**Figure 3:**
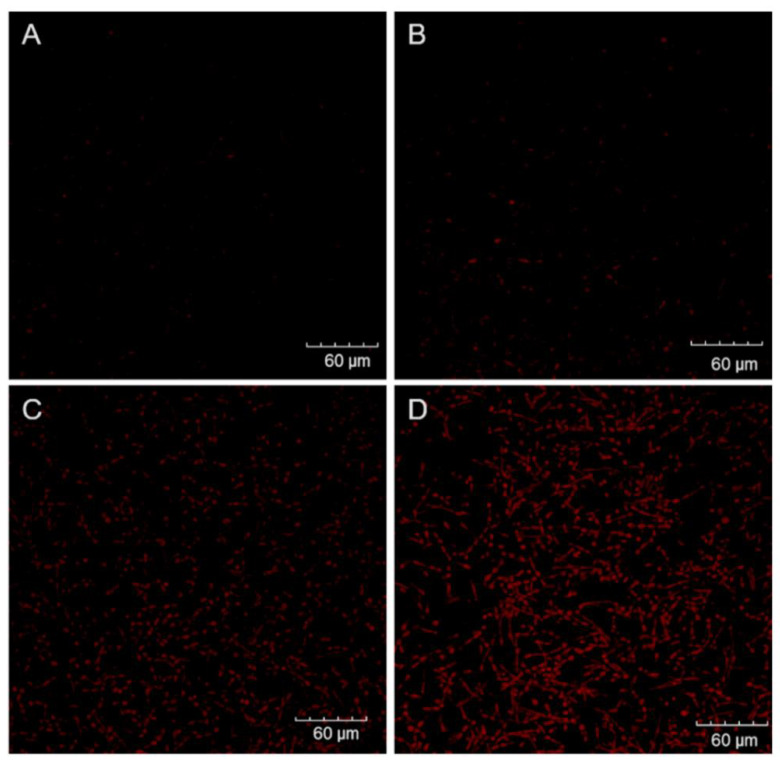
Representative confocal microscopy images of *C. albicans* ATCC 90028 biofilms grown for 48 h and stained with PI after treatments. Control (**A**): biofilm without irradiation; dark (**B**): biofilm incubated with 1.5 µM of ZnP hexyl without irradiation; light (**C**): biofilm irradiated only (4.3 J/cm^2^); aPDI (**D**): biofilm incubated with 0.8 µM of ZnP hexyl for 10 min followed by irradiation (4.3 J/cm^2^).

To further corroborate the MTT assay results, treated biofilms were analyzed by PI staining using confocal microscopy. As shown in Figure 3, no noteworthy fluorescence was detected from control or dark groups. In agreement with the MTT assay results, the light group displayed distinct PI labeling (Figure 3C), but it was not as intense or uniform as the red fluorescence signal in the aPDI group with ZnP hexyl at 0.8 µM (Figure 3D).

**Figure 4:**
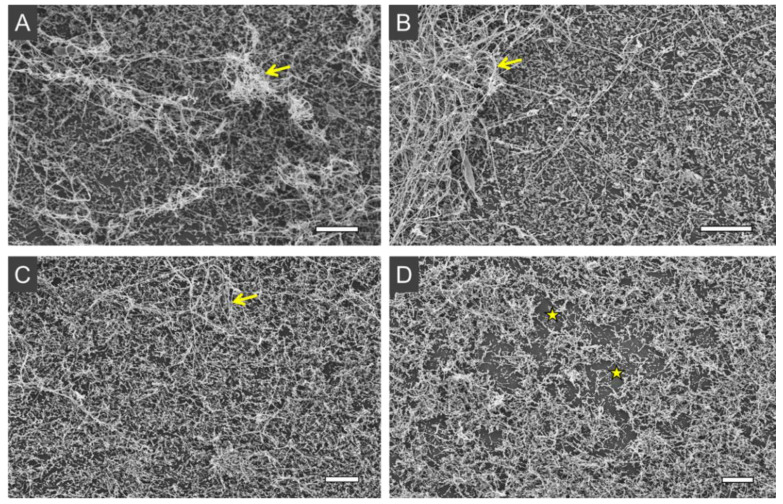
Representative SEM images of *C. albicans* ATCC 90028 biofilms grown for 48 h and submitted to different treatments. Control (**A**): biofilm without irradiation; dark (**B**): biofilm incubated with 1.5 µM of ZnP hexyl without irradiation; light (**C**): biofilm irradiated only (4.3 J/cm^2^); aPDI (**D**): biofilm incubated with 0.8 µM of ZnP hexyl for 10 min prior to irradiation (4.3 J/cm^2^). Yellow arrows indicate hyphae entanglements. Yellow stars indicate the exposed substrate.

Employing another imaging approach, the treated samples were assessed by SEM to investigate the effect of aPDI on the ultrastructure of biofilms. The 48 h biofilms showed enriched hyphae content (Figure 4A–C), with some of these filamentous structures forming thick tangles deposited on top of the spread yeast cells, completely covering the substrate. In turn, aPDI-treated samples presented some biofilm disorganization, with reduced hyphae tangles, and more substrate exposure (Figure 4D).

### 3.3. Cytotoxicity on Mammalian Cells

**Figure 5:**
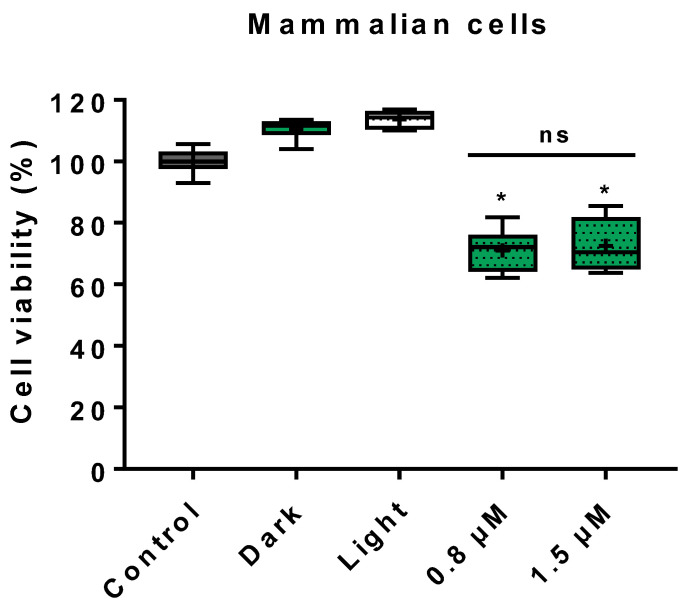
Box plot of cell viability of Vero cells assessed through MTT assay following treatment. Control: untreated cells; dark: cells incubated with 1.5 µM of ZnP without irradiation; light: cells irradiated in PBS only (4.3 J/cm^2^). Photodynamic treatment was performed for cells incubated with 0.8 or 1.5 µM of ZnP for 10 min followed by irradiation (4.3 J/cm^2^). The results were expressed in relation to untreated samples. Differences between groups were analyzed by Mann–Whitney test and considered significant at *p* < 0.05). At least three independent experiments were performed. *: *p* < 0.05 compared to control. ns: not significantly different, *p* > 0.05.

The assessment of the effect of ZnP hexyl-mediated photodynamic treatment was performed using Vero cells, a consolidated mammalian epithelial cell model (Figure 5). The results showed preserved viability for dark and light groups compared to control, and samples submitted to photodynamic treatment presented cell viability greater than 70% for both PS concentrations of 0.8 and 1.5 µM.

## 4. Discussion

### 4.1. Photoinactivation Effect on Yeast Cells

In this study, the effect of aPDI mediated by ZnP hexyl on *C. albicans* planktonic yeasts (ATCC 10231 and ATCC 90028) was investigated. Results showed no toxicity for the samples treated with PS in the dark or light alone. This is in agreement with previous studies, which also reported absence of dark toxicity of ZnP hexyl for other microorganisms, such as *Escherichia coli*, *S. cerevisiae*, and *Leishmania* spp. [21,25,34]. Regarding the photodynamically treated groups, differences in the susceptibility levels, as occurred in our study, have been reported across *C. albicans* isolates submitted to antimicrobial therapy [35], including aPDI [36,37].

ZnP hexyl-mediated aPDI has shown efficient results to photoinactivate a variety of microorganisms, including *E. coli*, *S. cerevisiae*, and *Leishmania* parasites [21,25,34]. It has been observed that the incorporation of a diamagnetic ion, i.e., Zn(II), in the center of the tetrapyrrole porphyrin ring greatly impacts the photophysical properties of PSs [10,38], and it can also improve their cellular uptake [24]. Moreover, the cationic, lipophilic, and amphiphilic properties of ZnP hexyl can provide a superior interaction of this Zn(II) porphyrin with the negatively charged cell surface, and contribute to its cellular uptake when compared to anionic compounds or more hydrophilic analogues [25]. Together, these characteristics can support the promising results herein obtained for aPDI of *C. albicans* yeast cells.

Only sparse studies have employed Zn(II) compounds to photoinactivate *C. albicans* [29,39,40,41]. In a previous work by our group, Viana et al. [29] reported the photoinactivation of *C. albicans* yeasts (ATCC 10231) mediated by the more hydrophilic analogue Zn(II) *meso*-tetrakis(*N*-ethylpyridinium-2-yl)porphyrin (ZnP ethyl). The authors obtained a 3 log_10_ reduction of CFU/mL after aPDI with blue light at 460 ± 20 nm (PIT: 10 min; light dose of ~81 J/cm^2^) with 10 µM of the PS. Although the previous work has used a light source different from the one used herein, it is possible to observe an improved photokilling activity for the ZnP hexyl analogue investigated in this present study. Considering the tetracationic character of both Zn(II) porphyrins, and that they only differ in the length of the aliphatic chain of their *meso* substituents, the higher lipophilicity of the ZnP hexyl (six-carbon chain) analogue enhances its cellular uptake [30], justifying its higher performance compared to ZnP ethyl (two-carbon chain).

Cormick et al. [19] investigated the importance of PS charge in the interaction with fungal cells. The authors compared the efficacy of cationic and anionic porphyrins at the same concentrations (1–5 µM) and irradiation parameters (PIT: 30 min; irradiation: 30 min–90 mW/cm^2^) against *C. albicans* and found that the positively charged PSs, 5-(4-trifluorophenyl)-10,15,20-tris(4-trimethylammoniumphenyl)porphyrin (TFAP^3+^) and 5,10,15,20-tetrakis(4-*N*,*N*,*N*-trimethylammoniumphenyl)porphyrin (TMAP^4+^) promoted better inactivation of *C. albicans* than the anionic PS 5,10,15,20-tetrakis(4-sulfonatophenyl)porphyrin (TPPS^4-^). At 5 μM, these cationic porphyrins exhibit a photosensitizing activity causing a ∼5 log_10_ decrease in cell survival. These results were attributed to the fact that the cationic PSs had higher binding affinity to fungal cells, which may be explained by the anionic character of fungal cell walls [19,42]. Another related *N*-alkylpyridiniumporphyrin-based PS, the tetracationic metal-free *meso*-tetrakis(*N*-methylpyridinium-4-yl)porphyrin (H_2_TM-4-PyP^4+^), mediated the complete inactivation of ATCC 10231 yeast cells by aPDI (fluorescent lamps, λ = 380–700 nm; 43.2 J/cm^2^) at 10 µM [43]. In addition to the longer aliphatic chain and enhanced lipophilicity, it is possible that the presence of the chelated Zn(II) in ZnP hexyl contributes to its increased performance compared to the free-base H_2_TM-4-PyP^4+^. Chelation of Zn(II) in the tetrapyrrole porphyrin ring has been shown to increase the triplet state lifetimes of *N*-alkylpyridinium porphyrins [44], which is associated with a higher potential of the PS to generate ROS [45].

Porphyrin derivatives have also been explored to mediate aPDI of the *C. albicans* strain ATCC 90028. Mang et al. [46] employed the clinically approved Photofrin^®^ at 25 µg/mL and a laser source (λ = 630 nm; 45 J/cm^2^) and observed a decrease in yeast survival by 92%. In another study, a similar commercial porphyrin, Photogem^®^, mediated complete photoinactivation of these yeasts at 50 µg/mL under blue light radiation (LED, λ = 450/10 nm; 18 J/cm^2^) [47]. It is, thus, worth noting that the ZnP hexyl-based aPDI protocol in this present study promoted inactivation of both *C. albicans* strains, while requiring lower concentrations (<1.5 µM or ~1.75 µg/mL) and milder irradiation parameters. This was also true when the results were compared with aPDI of these yeasts mediated by other PSs, such as dyes (methylene blue, indocyanine green, hypericin, rose Bengal, and malachite green oxalate) [48,49,50], other porphyrins [51,52], bacteriochlorin [53], and phthalocyanine [41].

### 4.2. Photoinactivation Effect on Biofilms

The ability of *C. albicans* to form biofilm communities, in which fungal cells are immersed into an ECM, is a major virulence and resistance factor, known to complicate microbial clearance by antifungal therapies [7]. A study by Garcia et al. [54] also confirmed the increased resistance that biofilms impose to aPDI, as they found that *C. albicans* mutant strains with deficient biofilm production were more susceptible to photoinactivation than a wild-type, biofilm-competent strain. *C. albicans* biofilms also tend to be rich in hyphae, a filamentous form of this fungus relevant for tissue invasion [7].

Our study also evaluated the effect of aPDI mediated by ZnP hexyl on these complex fungal forms. Results obtained from the group treated only with light revealed about 30% reduction in cell viability (Figure 2). This result was different from those reported by Ma et al. [16], in which blue light (LED λ = 455 nm; 13.2 J/cm^2^) alone had no noteworthy effect on ATCC 90028 biofilms. Blue light irradiation has been investigated as an antimicrobial treatment for *C. albicans* biofilms, but such studies usually applied higher light doses (~10 –50-fold higher) than the one used here [55,56,57]. In this case, endogenous photosensitizing molecules may be responsible for ROS generation and fungal killing [58]. We, however, believe that the presence of hyphae in biofilms might play a role in the results found here. It has been reported that hyphae forms, which are more abundantly in biofilms, show differences when compared to yeasts in the expression of targets of oxidative stress, such as the cell envelope components [59]. Moreover, hyphae have decreased levels of the antioxidant glutathione, increasing their susceptibility to oxidative stress [60]. The aPDI effect found in our study, however, was significantly more pronounced than that of blue light alone, reducing biofilm viability by ~89% (*p* < 0.05) at a submicromolar concentration of 0.8 µM.

*C. albicans* biofilms have been targeted by aPDI mediated by various PSs, including other porphyrins, photodithazine (PDZ, chlorin e6 derivative), protoporphyrin IX through 5-aminolevulinic acid (ALA) precursor, methylene blue, toluidine blue, erythrosine, and curcumin [15,16,17,37,61,62,63,64]. Shi et al. [61] incubated *C. albicans* biofilms for 5 h with 15 mM of ALA, followed by irradiation (laser, λ = 635 nm; 300 J/cm^2^), and found a biofilm inhibition of about 74.5%. Davies et al. [17] explored the metal-free porphyrin H_2_TM-4-PyP^4+^ at 7.3 µM as PS to photoinactivate *C. albicans* biofilms (PIT: 30 min; λ = 350–800 nm; 58.5 J/cm^2^). Using different strains than that of our study, ATCC MYA-274 and ATCC MYA-2732, only the latter showed susceptibility to the phototreatment, presenting cell viability reduction of 13.6%. In a study by Ma et al. [16], *C. albicans* biofilms were incubated for 20 min with curcumin at 60 µM prior to irradiation (LED, λ = 455 nm; *ca.* 7.9 J/cm^2^), and the authors found a decrease in the biofilm viability by 90.9%.

The aPDI was also investigated by acquiring confocal microscopy images of biofilms after incubation with PI, as well as by performing SEM analyses. PI is a cell-impermeant dye that only binds to the nucleic acids in cells with compromised membrane integrity, and does not accumulate effectively in viable cells [65]. The PI assay results corroborated the MTT viability analyses, showing a pronounced fluorescent labeling of aPDI-treated biofilms, compared to the other groups. This observation is in agreement with those found in other photodynamic studies with *C. albicans* biofilms [16,54,61,66]. Regarding SEM analyses, the images showed an effect on biofilm complexity, with fewer hyphae tangles, after aPDI. Such changes of biofilm structure promoted by aPDI have also been described in other studies [15,64]. It was not possible to observe the ECM in our biofilm samples, possibly due to the fixation process required for SEM, as also reported by Costa et al. [64] Using low-vacuum SEM, with no sample fixation requirement, Suzuki et al. [15] described a loss of ECM following aPDI of *C. albicans* biofilms with methylene blue, which could not be assessed by our approach.

Therefore, in the present study, we proposed a promising aPDI protocol based on ZnP hexyl to inactivate *C. albicans* yeasts and biofilms using short PIT, low PS concentration, and light dose. To the best of our knowledge, this is the first report of ZnP hexyl-aPDI against yeasts and biofilms of *C. albicans*.

### 4.3. Cytotoxicity on Mammalian Cells

The photodynamic effect on mammalian cells was also studied. Samples treated with PS in the dark or light alone, as well as photodynamically treated samples, showed cell viability greater than 70%. The observation of increased cell viability for Vero cells treated with light alone is not unusual, as it is known that blue light irradiation can exert regulatory effects on mitochondrial activity [67,68]. Regarding the higher percentual also found in the group treated with PS in the dark, we hypothesize that might be explained by the ZnP hexyl affinity to the mitochondria [31], which may also cause an effect on the metabolism of these organelles. As for the aPDI results, similar findings were reported by Andrade et al. [28] using Vero cells and the analogue ZnP ethyl, in which cell viability was about 70% for the two PS concentrations tested (5 and 10 µM). Souza et al. [21] treated bone marrow-derived macrophages with ZnP hexyl-mediated aPDI, using the same light source of the present study (*ca.* 3.4 J/cm^2^), and also found high levels of cell viability (around 80%) for both 0.62 and 1.25 μM concentrations of PS. It is also relevant to note that PDT is a local therapy, and its effects are focused on the site of administration of the PS and irradiation, which minimizes effects on healthy host tissue. This, coupled with our results showing controlled toxicity on mammalian cells in vitro, support the potential of ZnP hexyl-mediated aPDI to treat superficial forms of *C. albicans* infections.

## 5. Conclusions

Considering the relevance of *C. albicans* as a human pathogen, the challenges related to biofilm formation and the emergence of resistance, the search for alternative antifungal approaches has been encouraged. The present study reported a promising in vitro photoinactivation of *C. albicans* yeasts and biofilms using ZnP hexyl-mediated aPDI applying low PS concentrations and mild irradiation parameters. Our results stimulate further investigations using this PS in aPDI of other *Candida* species and resistant isolates.

## Data Availability

The data generated during this study are included in this article and in its supporting information openly available in Zenodo at https://doi.org/10.5281/zenodo.6564350.
